# Supplementary material for: A synthetic planar cell polarity system reveals localized feedback on Fat4-Ds1 complexes
Source: eLife. 2017 Aug 18;6:e24820. doi: 10.7554/eLife.24820 (PMC5576920; doi:10.7554/eLife.24820)
Supplement: Transparent reporting form [file elife-24820-transrepform.docx]

***eLife’s* transparent reporting form**

We encourage authors to provide detailed information *within their submission* to facilitate the interpretation and replication of experiments. If you have any questions, please contact us: [editorial@elifesciences.org](mailto:editorial@elifesciences.org).

**Sample-size estimation**

- You should state whether an appropriate sample size was computed when the study was being designed
- You should state the statistical method of sample size computation and any required assumptions
- If no explicit power analysis was used, you should describe how you decided what sample (replicate) size (number) to use

Please outline where this information can be found within the submission (e.g., page numbers or figure legends), or explain why this information doesn’t apply to your submission:

Fig2: For each condition we took 10 images with ~10^3 cells/ image. This to make the std error in the mean of a time point less than the typical difference in the mean of the fraction of accumulating boundaries between time points, i.e. ~ 0.02 (Figure 2 –supplementary 1). Number of images and mean number of cells are stated in the figure.

Similar number were used for the second dataset (Figure 2 – figure supplement 2)

Fig3: DE sample sizes were not determined before the analysis, but are more than enough to establish significance, with n > 30 and ρ = 0.99, p << 10^-5^. n and ρ are stated in the figure.

Fig 4 EF: sample sizes for boundary FRAP were chosen to be ~10 to convincingly show significance of the difference between complexes and unbound proteins dynamics (since the difference is more than two orders of magnitude).

Sample size for the FRAP experiment on unbound Fat4, Ds1, N-cad, was chosen to allow distinguishing between the mean rate of diffusion of these proteins. Number of experiments are provided in the caption.

Fig 5. Sample size was chosen so that even if treating each boundary as a single measurment (in reality it constitutes many independent measurements), the std error in the mean gap would be less than 10nm. Sample sizes and std are in the figure.

**Replicates**

- You should report how often each experiment was performed
- You should include a definition of biological versus technical replication
- The data obtained should be provided and sufficient information should be provided to indicate the number of independent biological and/or technical replicates
- If you encountered any outliers, you should describe how these were handled
- Criteria for exclusion/inclusion of data should be clearly stated
- High-throughput sequence data should be uploaded before submission, with a private link for reviewers provided (these are available from both GEO and ArrayExpress)

Please outline where this information can be found within the submission (e.g., page numbers or figure legends), or explain why this information doesn’t apply to your submission:

Fig 2: Within each timepoint in the experiment, 10 independent images with ~1000 cells in each image were collected. A biological duplicate of this experiment largely reproduced the results (provided in figure 2 – supplement figure 2)

Fig 3. Experiments C and D were each repreated 3 times with 2 repeats shown in Figure 3 - figure supplement 1.

**Statistical reporting**

- Statistical analysis methods should be described and justified
- Raw data should be presented in figures whenever informative to do so (typically when N per group is less than 10)
- For each experiment, you should identify the statistical tests used, exact values of N, definitions of center, methods of multiple test correction, and dispersion and precision measures (e.g., mean, median, SD, SEM, confidence intervals; and, for the major substantive results, a measure of effect size (e.g., Pearson's r, Cohen's d)
- Report exact p-values wherever possible alongside the summary statistics and 95% confidence intervals. These should be reported for all key questions and not only when the p-value is less than 0.05.

Fig 2D. limits in figure indicate 95% confidence interval.

Fig 3DE. Pearson correlation stated in figure.

Fig 4EF. significance was tested using t-test and p-values are stated in the caption.

Please outline where this information can be found within the submission (e.g., page numbers or figure legends), or explain why this information doesn’t apply to your submission:

(For large datasets, or papers with a very large number of statistical tests, you may upload a single table file with tests, Ns, etc., with reference to page numbers in the manuscript.)

**Additional data files (“source data”)**

- We encourage you to upload relevant additional data files, such as numerical data that are represented as a graph in a figure, or as a summary table
- Where provided, these should be in the most useful format, and they can be uploaded as “Source data” files linked to a main figure or table
- Include model definition files including the full list of parameters used
- Include code used for data analysis (e.g., R, MatLab)
- Avoid stating that data files are “available upon request”

Please indicate the figures or tables for which source data files have been provided:

For fig 2 the analysis code is in <https://github.com/idse/FatDs/tree/master/snapshot>.

Source data for figure 2 (sheet 1) and figure2 – supplement figure 2 (sheet 2) is available in alldata.xlsx
